# Interim analysis of a phase I/IIa trial assessing E39+GM-CSF, a folate binding protein vaccine, to prevent recurrence in ovarian and endometrial cancer patients

**DOI:** 10.18632/oncotarget.13305

**Published:** 2016-11-11

**Authors:** Doreen O. Jackson, Kevin Byrd, Timothy J. Vreeland, Diane F. Hale, Garth S. Herbert, Julia M. Greene, Erika J. Schneble, John S. Berry, Alfred F. Trappey, Guy Travis Clifton, Mark O. Hardin, Jonathan Martin, John C. Elkas, Thomas P. Conrads, Kathleen M. Darcy, Chad A. Hamilton, George L. Maxwell, George E. Peoples

**Affiliations:** ^1^ Department of Surgery, San Antonio Military Medical Center, San Antonio, TX, USA; ^2^ National Capital Consortium Fellowship in Gynecologic Oncology, Walter Reed National Military Medical Center Bethesda, MD, USA; ^3^ Gynecologic Cancer Center of Excellence, Annandale, VA, USA; ^4^ Department of Surgery, Womack Army Medical Center, Fayetteville, NC, USA; ^5^ Department of Surgical Oncology, The University of Texas MD Anderson Cancer Center, Houston, TX, USA; ^6^ Department of Surgery, Madigan Army Medical Center, Tacoma, WA, USA; ^7^ Cancer Vaccine Development Program, San Antonio, TX, USA; ^8^ Department of Obstetrics and Gynecology, Inova Fairfax Hospital Annandale, VA, USA; ^9^ Mid-Atlantic Gynecologic Oncology and Pelvic Surgical Associates, Annandale, VA, USA; ^10^ Inova Schar Cancer Institute, Inova Health System, Annandale, VA, USA

**Keywords:** cancer, ovarian, endometrial, folate binding protein, immunotherapy

## Abstract

**BACKGROUND:**

Folate binding protein(FBP) is an immunogenic protein over-expressed in endometrial(EC) and ovarian cancer(OC). We are conducting a phase I/IIa trial of E39 (GALE 301)+GM-CSF, an HLA-A2-restricted, FBP-derived peptide vaccine to prevent recurrences in disease-free EC and OC patients. This interim analysis summarizes toxicity, immunologic responses, and clinical outcomes to date.

**METHODS:**

HLA-A2+ patients were vaccinated(VG), and HLA-A2- or -A2+ patients were followed as controls(CG). Six monthly intradermal inoculations of E39+250mcg GM-CSF were administered to VG. Demographic, safety, immunologic, and recurrence rate(RR) data were collected and evaluated.

**RESULTS:**

This trial enrolled 51 patients; 29 in the VG and 22 in the CG. Fifteen patients received 1000mcg E39, and 14 received <1000mcg. There were no clinicopathologic differences between groups(all *p* = 0.1). E39 was well-tolerated regardless of dose. DTH increased pre- to post-vaccination (5.71.5 mm vs 10.33.0 mm, *p* = 0.06) in the VG, and increased more in the 1000mcg group (3.82.0 mm *vs* 9.53.5 mm, *p* = 0.03). With 12 months median follow-up, the RR was 41% (VG) *vs* 55% (CG), *p* = 0.41. Among the 1000mcg patients, the RR was 13.3% vs 55% CG, *p* = 0.01. Estimated 2-year DFS was 85.7% in the 1000mcg group vs 33.6% in the CG (*p* = 0.021).

**CONCLUSIONS:**

This phase I/IIa trial reveals that E39+GM-CSF is well-tolerated and elicits a strong, dose-dependent *in vivo* immune response. Early efficacy results are promising in the 1000 mcg dose cohort. This study proves the safety and establishes the dose of E39 for a larger prospective, randomized, controlled trial in HLA-A2+ EC and OC patients to prevent recurrence.

## INTRODUCTION

Endometrial cancer is the most common gynecologic cancer in the United States [1]. Ovarian cancer, though less common, is the most common cause of death amongst gynecologic cancers [2]. Due to the nonspecific symptoms of ovarian cancer, > 70% of patients will present with late stage disease [2] and, even with aggressive regimens, fewer than 40% of women with ovarian cancer are cured after standard of care treatment [2]. Endometrial cancer has a much better prognosis in general because of earlier diagnosis [1], but even in early stage disease, 10-15% of patients will experience a recurrence. The serous variant of endometrial cancer is particularly aggressive, often presenting with evidence of metastasis [3] and having a clinical course more like that of ovarian cancer than typical endometrial cancer. Although only responsible for 3-10% of all endometrial cancers, this subtype leads to a high risk of recurrence, and up to 40% of endometrial cancer deaths [3]. Current treatments for endometrial and ovarian cancer include surgery, chemotherapy, and radiation, but these are insufficient in many patients, particularly those with unfavorable histology and advanced tumor stages.

Due to the high recurrence rates, patients who present with International Federation of Gynecology and Obstetrics (FIGO) Stage II or higher endometrial and ovarian cancers are commonly referred to participate in clinical trials investigating new therapies. One such trial, a recent phase III trial, evaluated the efficacy of a prolonged chemotherapy regimen in Stage III and IV ovarian cancer patients who were randomized to either 3 or 12 cycles of paclitaxel. This trial did show a significant increase in progression free survival (*p* = 0.006), but no difference in overall survival. In addition, this trial included a high dropout rate due to significant neurotoxicity from prolonged chemotherapy [4]. Given these disappointing results with even extended chemotherapy, novel medications have also been developed, including those that inhibit biochemical processes (anti-angiogenesis, DNA repair mechanisms, mTOR pathway, etc.) and monoclonal antibodies (anti-CA125 and anti-human epithelial cell adhesion molecule-EpCAM) [5, 6]. Despite some promising work with these various regimens, durability of response remains limited. Given the propensity for recurrence in this setting, a treatment with better long-term efficacy is needed. Active immunity induced by a successful peptide vaccine offers the promise of a tumor-specific T cell response, generating immunologic memory that could potentially lead to prolonged protection from tumor recurrence.

Our group has had success inducing such active immunity with peptide vaccines in breast cancer patients. We have developed a peptide vaccine, E75 or NeuVax (nelipepimut-S), which is directed at the well-known tumor associated antigen (TAA), HER2. A phase I/II trial studying NeuVax in patients with node positive and high-risk, node negative breast cancer has recently been completed. The vaccine was well tolerated, with minimal toxicity, and successfully induced a significant immunologic response to HER2, particularly in patients receiving the optimal dose. As a result of this response, patients who received the optimal dose of the vaccine had an estimated 5-year disease free survival (DFS) of 94.6% compared to 80.2% for the prospectively followed controls (*p* = 0.059) [7]. This vaccine is now being studied in a phase III trial.

Building on the success of NeuVax, we sought to identify a TAA that could be targeted by a similar peptide vaccine in endometrial and ovarian cancer. The ideal TAA is highly expressed in malignant cells, with minimal to no expression in normal tissue, and correlates with aggressive disease. The folate binding protein (FBP), also known as folate receptor-alpha, is a TAA common to both endometrial and ovarian cancer [8] with up to 80-90 fold higher expression in malignant cells compared to normal cells [8, 9]. FBP levels coincide with increased folate uptake, which is necessary for DNA production in rapidly replicating cells [10]. As a result, its over-expression has been associated with higher grade and stage of endometrial [1] and ovarian cancers [10]. High levels of FBP have also been associated with the more aggressive serous variant of endometrial cancer [11]. Furthermore, FBP may also be associated with failure to respond to platinum-based chemotherapy and reduced survival in ovarian cancer patients with residual disease after surgical treatment [12]. Highlighting the importance of this TAA, there have already been significant efforts toward developing novel FBP-targeted therapies, including a monoclonal antibody, farletuzumab, and two multi-peptide vaccines that are in the early stages of development.

E39 (GALE 301) is an HLA-A2 restricted, FBP-derived (191-199, EIWTHSYKV ) [13, 14] peptide, which was shown to enhance tumor-associated lymphocyte proliferation and anti-tumor function [15] in pre-clinical trials. We are conducting a prospective phase I/IIa trial of E39+ granulocyte macrophage-colony stimulating factor (GM-CSF) to prevent recurrence in disease-free endometrial and ovarian cancer patients at high risk for recurrence after standard of care treatments. Here, we present an interim analysis of the toxicity, *in vivo* immunologic responses, and current DFS in this trial.

## RESULTS

### Patients

**Figure 1:**
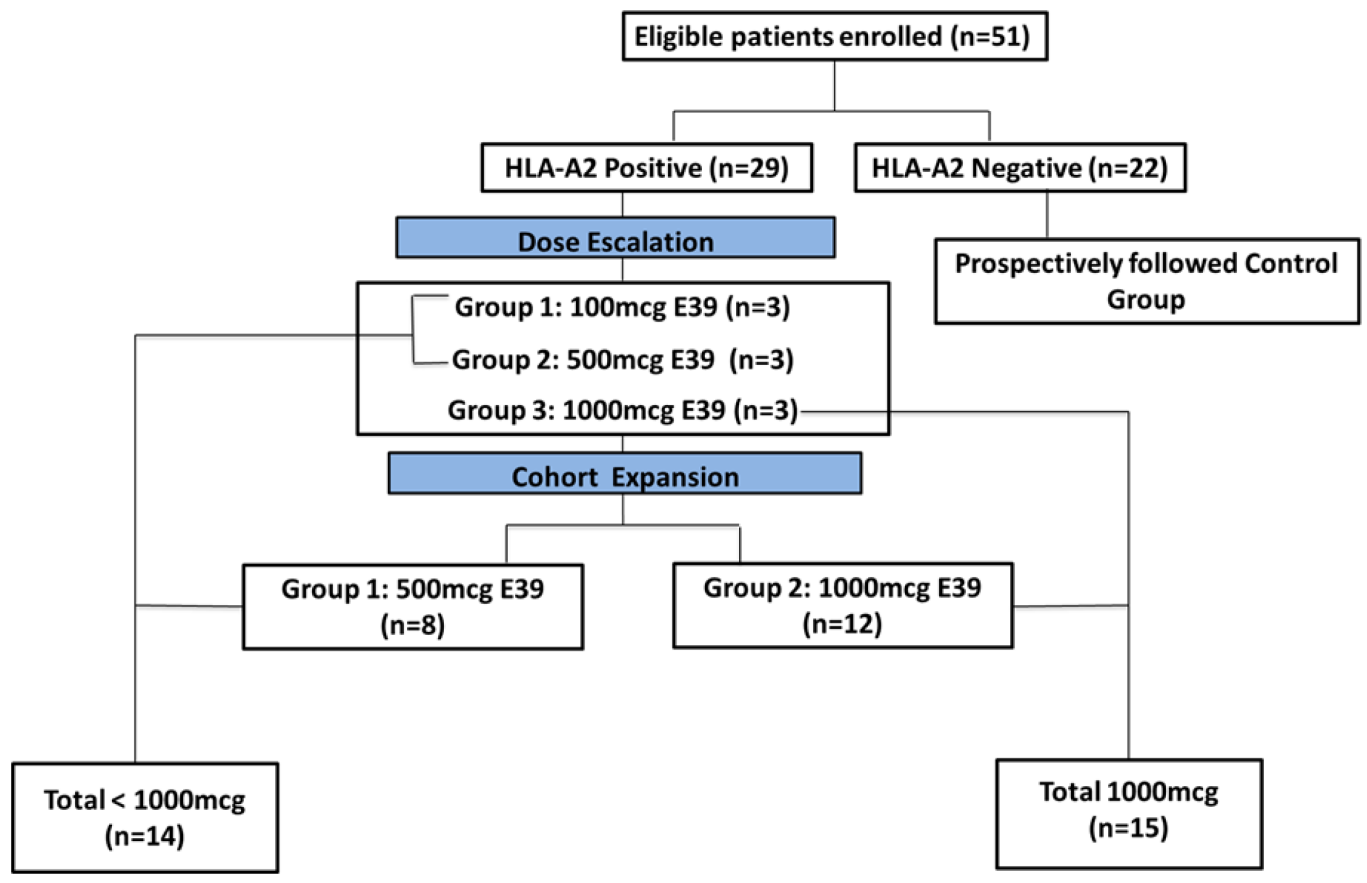
Patient flow for study enrollment The prospectively followed control group also included three HLA-A2 positive patients who declined vaccine administration.

We enrolled 51 patients, 29 in the vaccinated group (VG) and 22 in the control group (CG). Of the 51 patients, 40 patients were enrolled after standard of care treatment of primary disease (24 in the VG, 16 in the CG), and 11 patients after treatment of recurrent disease (5 in the VG, 6 in the CG). All patients were disease-free at enrollment. Within the VG, 15 patients received 1000 mcg of the peptide, while 14 patients received less than 1000 ( < 1000) mcg of E39. Refer to Figure 1 for the consort diagram. There were no significant clinicopathologic differences between groups (Table 1 and 2).

**Table 1:** Demographics

Characteristic	Vaccinated (*n*=29)	Controls (*n*=22)	*p*-Value
**Median age (yrs)**	60	61	0.790
(Interquartile Range 1-3)	52-67	53-63	
**Histology - *n* (%)**			0.373
Endometrial	6 (20.7)	3 (13.6)	
Fallopian	1 (3.4)	0 (0.0)	
Ovarian	20 (69.0)	18 (86.4)	
Peritoneal	2 (6.9)	0 (0.0)	
**Grade - *n* (%)**			0.851
1 (Well Differentiated)	2 (6.9)	2 (9.1)	
2 (Moderately Differentiated)	4 (13.8)	2 (9.1)	
3 (Poorly Differentiated)	23 (79.3)	18 (81.8)	
**T Stage - *n* (%)**			0.536
Tis	1 (3.4)	0 (0.0)	
1	6 (20.7)	3 (13.6)	
> 1	21 (72.4)	19 (86.4)	
Tx	1 (3.4)	0 (0.0)	
**Node - *n* (%)**			0.764
Positive	9 (31.0)	5 (22.7)	
Negative	20 (69.0)	17 (77.3)	
**FIGO Stage - *n* (%)**			0.591
I	4 (13.8)	3 (13.6)	
II	2 (6.9)	3 (13.6)	
III	18 (62.1)	11 (50.0)	
IV	5 (17.2)	5 (22.7)	
**Disease Status at enrollment - *n* (%)**			0.388
Primary	24 (82.8)	16 (72.7)	
Recurrent	5 (17.2)	6 (27.3)	

**Table 2:** Demographics – Dosing Cohort

Characteristic	Vaccinated <1000mcg (*n*=14)	Vaccinated 1000mcg (*n*=15)	Controls (*n*=22)	*p*-Value
**Median age (yrs)**	61	57	61	0.827
(Interquartile Range 1-3)	56 - 66	49 - 67	53 - 63	
**Histology – *n* (%)**				0.531
Endometrial	3 (21.4)	3 (20.0)	3 (13.6)	
Fallopian	1 (7.1)	0 (0.0)	0 (0.0)	
Ovarian	9 (64.3)	11 (73.3)	18 (86.4)	
Peritoneal	1 (7.1)	1 (6.7)	0 (0.0)	
**Grade – *n* (%)**				0.508
1 (Well Differentiated)	0 (0.0)	2 (13.3)	2 (9.1)	
2 (Moderately Differentiated)	3 (21.4)	1 (6.7)	2 (9.1)	
3 (Poorly Differentiated)	11 (78.6)	12 (80.0)	18 (81.8)	
**T Stage – *n* (%)**				0.469
Tis	0 (0.0)	1 (6.7)	0 (0.0)	
1	3 (21.4)	3 (20.0)	3 (13.6)	
> 2	11 (78.6)	10 (66.7)	19 (86.4)	
Tx	0 (0.0)	1 (6.7)	0 (0.0)	
**Node – *n* (%)**				0.450
Positive	6 (42.9)	3 (20.0)	5 (22.7)	
Negative	8 (57.1)	12 (80.0)	17 (77.3)	
**FIGO Stage – *n* (%)**				0.297
I	1 (7.1)	3 (20.0)	3 (13.6)	
II	0 (0.0)	2 (13.3)	3 (13.6)	
III	11 (78.6)	7 (46.7)	11 (50.0)	
IV	2 (14.3)	3 (20.0)	5 (22.7)	
**Disease Status at enrollment – *n* (%)**				0.305
Primary	13 (92.9)	11 (73.3)	16 (72.7)	
Recurrent	1 (7.1)	4 (26.7)	6 (27.3)	

### Toxicity

**Figure 2:**
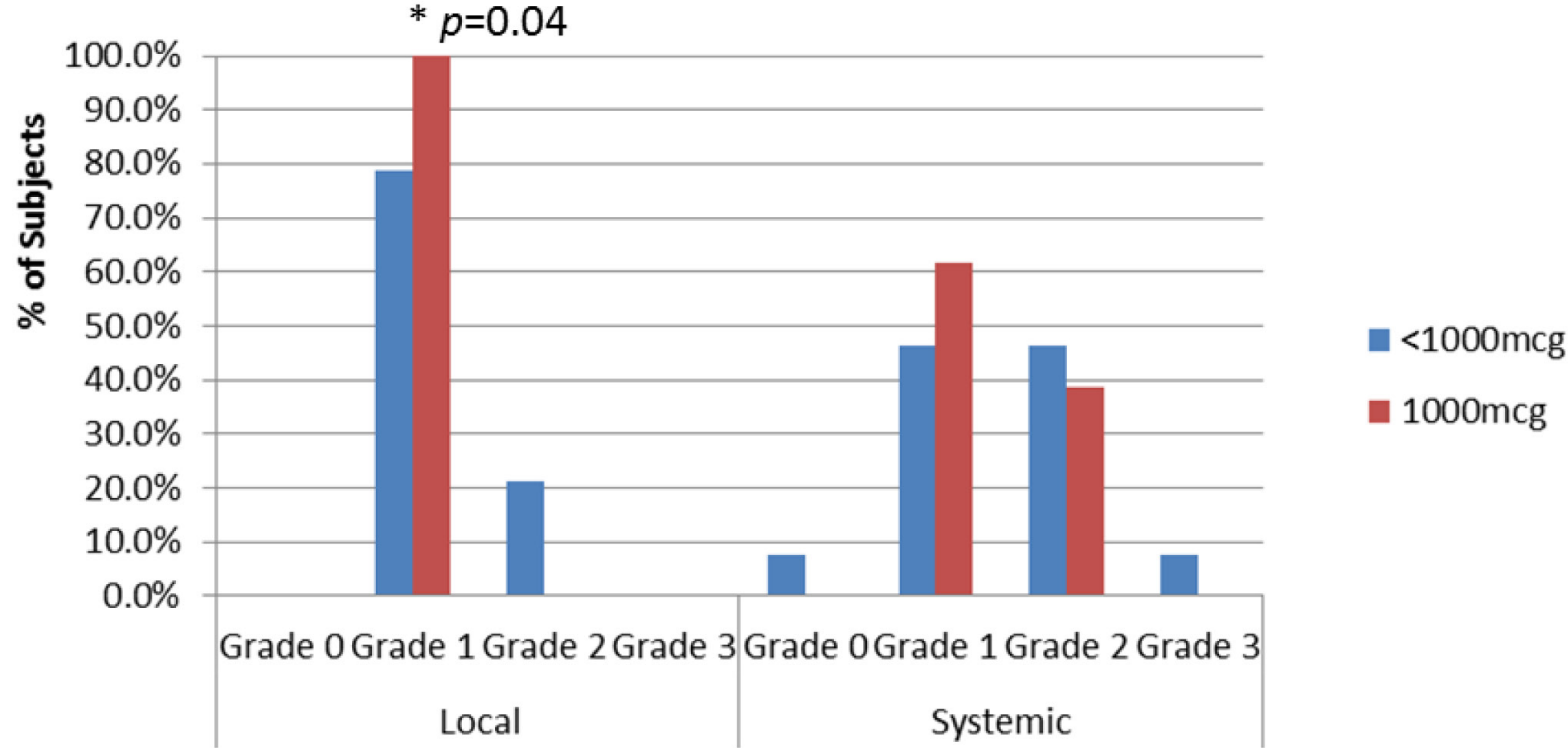
Maximum local and systemic toxicities appreciated within the dosing cohorts There was a statistically significant difference between the local toxicity experienced by the 1000mcg vs <1000mcg group (*p*=0.04). No significant difference was noted within systemic toxicity. This difference may be due to an increased consumption of GM-CSF with higher doses of the peptide, thus leading to a smaller amount of GM-CSF to cause worsened local or systemic toxicity.

Local and systemic toxicities were mild at the completion of the PVS (Figure 2), with no grade 4 or 5 toxicities, and only 1 patient experiencing grade 3 toxicity. The maximum local toxicities were milder for the 1000mcg patients than the < 1000mcg patients (*p* = 0.04) (100% *vs* 78.6% grade 1 and 0% *vs* 21.4% grade 2, respectively). The most common local toxicities were induration at the injection site, erythema, and pruritus. The maximum systemic toxicities for the 1000mcg group as compared to the < 1000mcg group were 0% *vs* 7.7% grade 0, 61.5% *vs* 46.2% grade 1, 38.5% *vs* 46.2% grade 2, and 0% *vs* 7.7% grade 3, respectively (*p* > 0.05 for each). The most common systemic toxicities were myalgias, headache, and fatigue (Figure 2).

### Immunologic response

**Figure 3:**
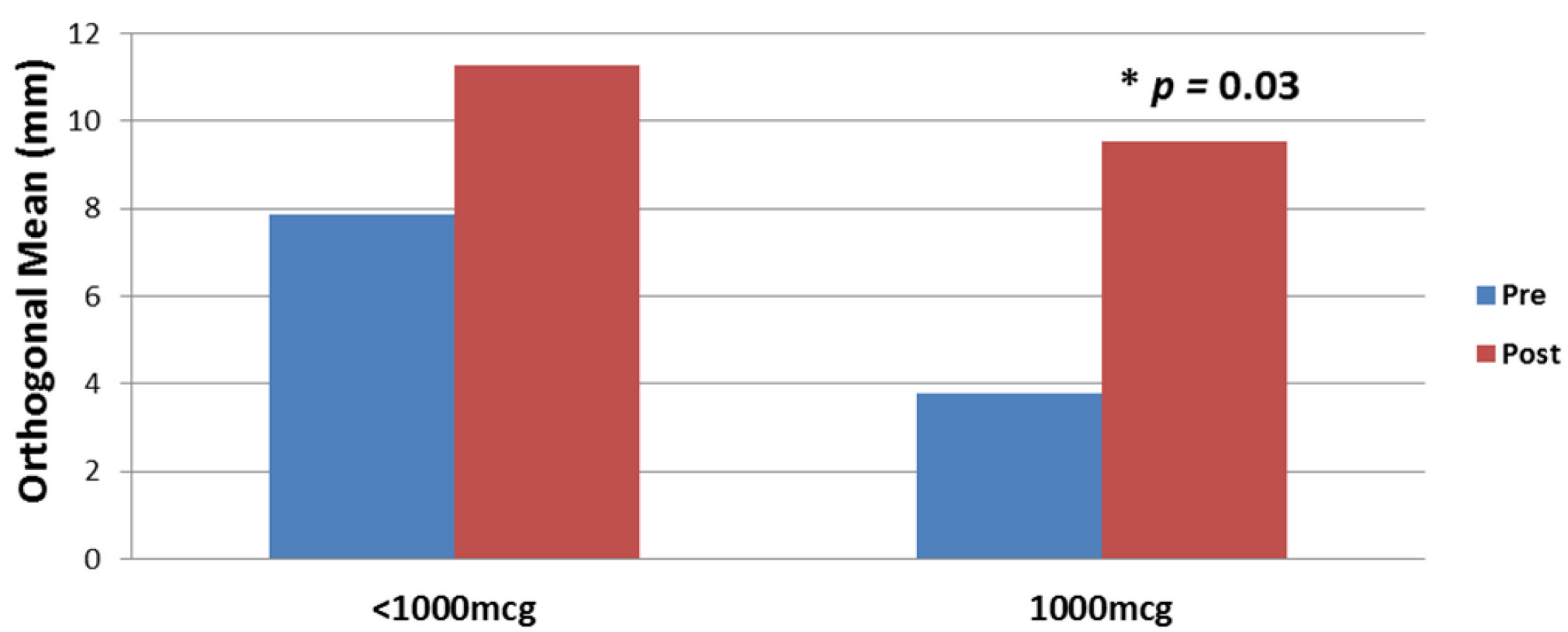
Immune response before and after the primary vaccination series according to dosing cohorts The average size DTH reaction in all vaccinated patients prior to vaccination was 5.7 ±1.5 mm compared to 10.3 ±2.8 mm post-vaccination (*p*=0.06). The 1000mcg patients had a statistically significant increase in pre-vaccination versus post-vaccination DTH (3.8 +2.0 mm vs 9.5 +3.5 mm, *p*=0.03), while <1000mcg patients experienced a smaller increase in pre- vs post-vaccination, which was not statistically significant (7.8 +2.1 mm vs 11.3 +4.8 mm, *p*=0.28).

The DTH increased from pre-vaccination to post-vaccination in the VG, approaching statistical significance (5.7±1.5 mm *vs* 10.3 ±2.8 mm, *p* = 0.06). As shown in Figure 3, when analyzed by dose, 1000mcg patients had a statistically significant increase in DTH from pre-vaccination to post-vaccination (3.8 +2.0 mm *vs* 9.5+3.5 mm, *p* = 0.03), while < 1000mcg patients experienced a smaller increase from pre- to post-vaccination, which was not statistically significant (7.8+2.1 mm *vs* 11.3+4.8 mm, *p* = 0.28).

### Disease-free survival

**Figure 4:**
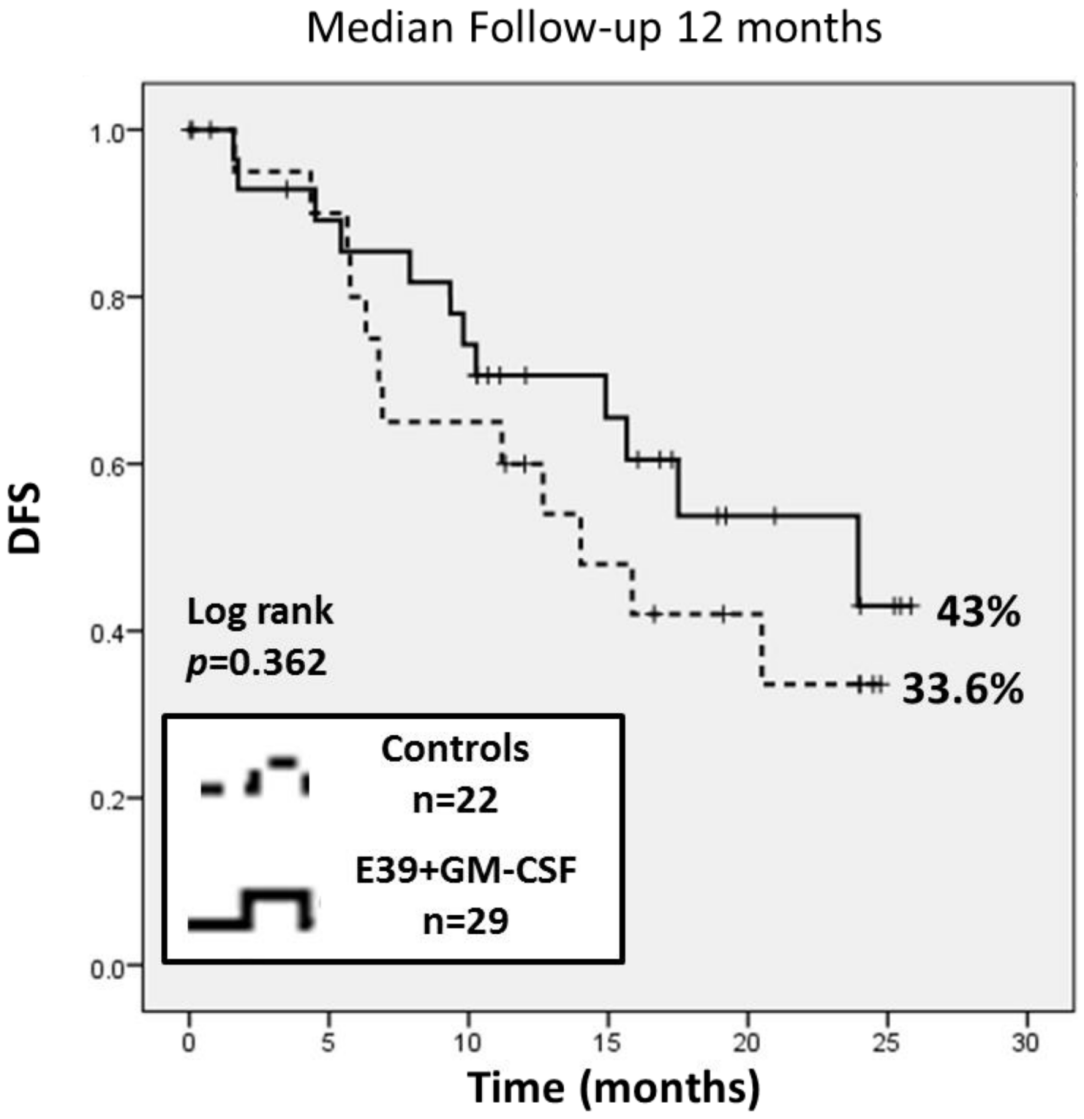
The 2-year estimated DFS for vaccinated patients was 43% (95% confidence interval (CI): 18-66%) versus 33.6% (95% CI: 13-56%) in control patients (p=0.36) The vaccinated patients experienced a 31% reduction in relative recurrence risk regardless of dose.

This planned interim analysis was performed 12 months after completion of trial enrollment. The median follow up was 12.0 months (interquartile range 7.6-19.2 months). The overall recurrence rate (RR) for the VG *vs* the CG was 41.4% *vs* 54.6%, respectively (*p* = 0.35). The 2-year estimated DFS for VG was 43% (95% confidence interval (CI): 18-66%) *vs* 33.6% (95% CI: 13-56%) in CG (*p* = 0.36). The vaccinated patients experienced a 31% reduction in relative recurrence risk regardless of dose (Figure 4).

**Figure 5:**
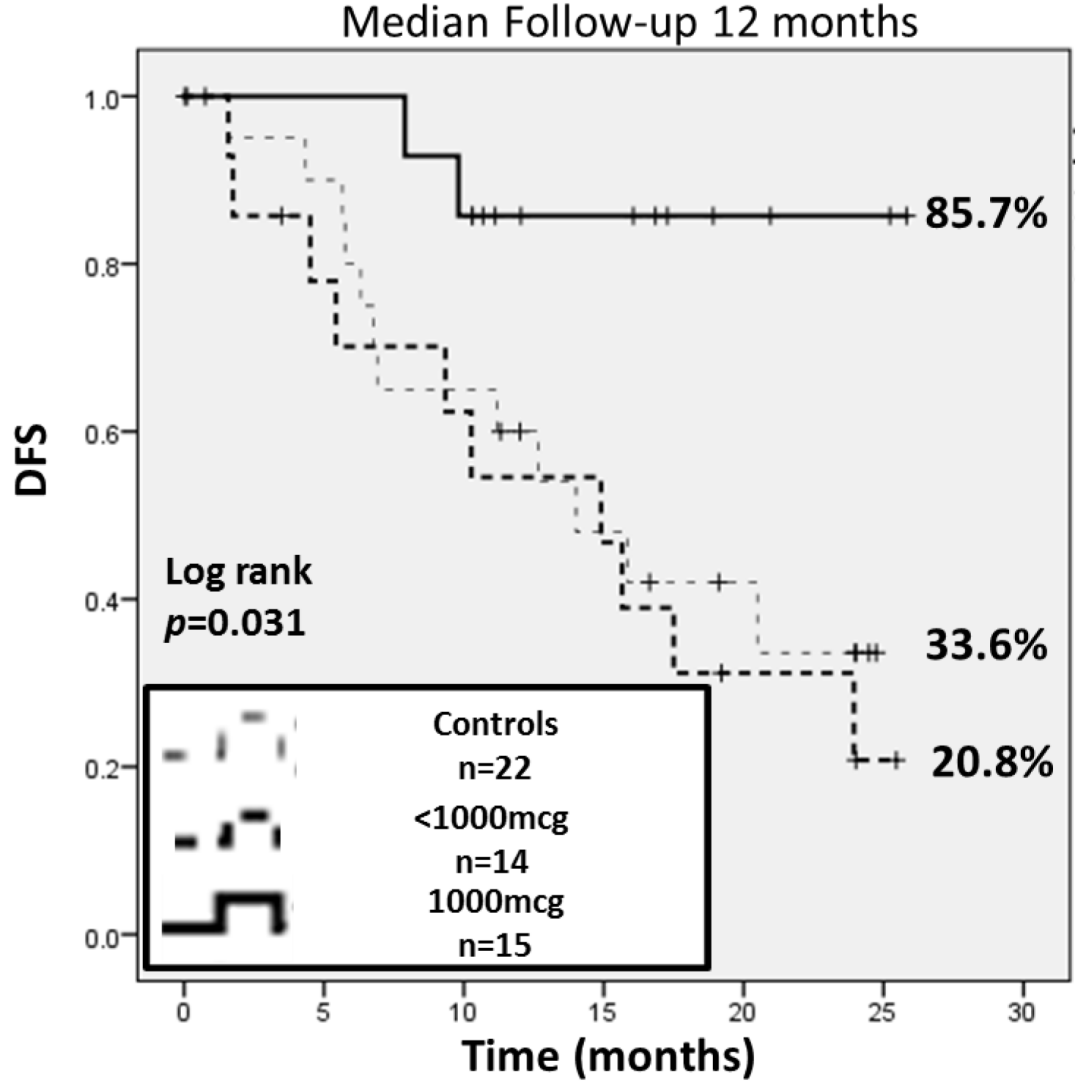
This subgroup analysis was performed based on dosing The 2-year estimated DFS indicated a significant survival advantage for the 1000 mcg group at 85.7% (95% CI: 54-96%) compared to controls at 33.6% (95% CI: 13-56%) and the <1000 mcg group at 20.8% (95% CI: 4-47%). Comparing the 1000 mcg and control groups, there was an 83% reduction in relative risk of recurrence.

Per protocol design, planned subgroup analyses were performed based on dosing cohort. The recurrence rate was significantly lower in the 1000mcg group compared to the CG patients (13.3% *vs* 54.6%, *p* = 0.02). There was no statistically significant difference in recurrence between the < 1000mcg group (71.4%) *vs* the CG patients. When comparing the dosing cohorts, patients receiving the 1000mcg dose of E39 experienced an 83% reduction in relative risk of recurrence compared to < 1000mcg patients (HR 0.17, 95% CI: 0.04 - 0.77, *p* = 0.003). The 2-year estimated DFS indicated a significant survival advantage for the 1000mcg group at 85.7% (95% CI: 54-96%) compared to the CG at 33.6% (95% CI: 13-56%, *p* = 0.02). There was no difference of statistical significance in the estimated 2-year DFS between the < 1000mcg group and CG. A survival benefit was appreciated with analysis of the estimated 2-year DFS comparing 1000mcg to the < 1000mcg patients, 85.7% *versus* 20.8% (*p* = 0.009). Figure 5 displays the survival analysis of a 3-way comparison between 1000mcg group, < 1000mcg group and CG.

An additional subset analysis was completed comparing patients with primary disesase at the time of enrollment to those with recurrent disease. Overall, 17.2% of VG and 27.2% of CG had recurrent disease. When analyzing the patients with recurrent disease only, the reccurrence rates were very similar between the VG and CG (60.0% *vs* 66.7%, *p* = 0.65). For primary disease patients, however, the RR was 41.6% and 50.0% in VG and CG, respectively (*p* = 0.42). Further comparisons of these groups based upon dosing were completed. The statistically significant survival benefit observed in the 1000mcg group remained significant for patients with primary disease, VG DFS 66.7% (95% CI: 5-95%) *vs* CG 36.7% (95% CI: 11-63%), *p* = 0.02. This relationship lost its significance, however, when applied to the 1000 mcg patients with recurrent disease, VG DFS 33.3% (95% CI: 1-77%) *vs* CG 22.2% (95% CI: 1-61%), *p* = 0.96.

## DISCUSSION

This study represents the first phase I/IIa clinical trial of a single peptide vaccine directed at FBP in endometrial and ovarian cancer. The interim analysis of this trial using E39+GM-CSF to prevent recurrence in disease-free endometrial and ovarian cancer patients at high risk of recurrence demonstrated that the vaccine is well tolerated, immunogenic and offers promising clinical efficacy.

FBP has been identified previously as an ideal target for immunotherapy based on its high expression in ovarian, endometrial and breast cancer cells, low expression in normal cells, and its association with aggressive disease [18]. Specifically, in endometrial cancer, the more aggressive serous variant has been linked to high levels of FBP expression. Underscoring the significant potential of FBP as a target of immunotherapy, several novel treatments aimed at this TAA have been developed. Some of these treatment strategies have included FBP targeted antibody-drug conjugates and folate bound toxins/drugs, but more relevant for this study are the attempts at active and passive immunity [18]. In addition to our development of E39, a monoclonal antibody treatment and two multi-peptide vaccines have been studied in clinical trials [22, 23, 24].

Farletuzumab (MOrAb-003), a humanized FBP monoclonal antibody, was evaluated in a recently completed phase III clinical trial evaluating its safety and efficacy [22]. In this trial, 1100 women with recurrent ovarian cancer after platinum-taxane chemotherapy were treated with carboplatin plus paclitaxel or docetaxel combined with either placebo, 1.25mg/kg of farletuzumab, or 2.5mg/kg of farletuzumab. The safety analysis of this trial revealed no differences between groups, confirming the safety of targeting FBP given its low expression in normal tissues. The overall progression-free survival (PFS) results from this trial showed no statistically significant difference between groups, but pre-specified subgroup analysis did show that, in patients with CA-125 levels less than three times the upper limit of normal, patients treated with 2.5mg/kg of farletuzumab had increased PFS and OS over patients receiving placebo. The authors suggest that the extremely high levels of CA-125 may block the immune effects of farletuzumab (*via* antibody-dependent cellular cytotoxicity, or ADCC). Additional findings showed that patients with higher serum levels of the drug (regardless of dose received) had improved results, suggesting that the pharmacodynamics of the drug are not well understood and a higher dose may be indicated in future trials [22]. While the primary endpoint of the trial was not reached in these heavily pre-treated and recurrent patients, farletuzumab remains a promising agent and FBP remains a very attractive target. Additionally, the possible immune mechanism of farletuzumab, much like trastuzumab in HER2-positive breast cancer, makes combination with an FBP-directed vaccine an attractive option for the future, particularly given the favorable toxicity profiles of both agents.

The two multi-peptide vaccines directed at FBP target a wide range of epitopes, aiming to induce an immune response to each. Similar to E39, these vaccines were designed to induce meaningful immune memory, which should help decrease disease recurrence. The first multi-peptide vaccine, which incorporated five immunogenic FBP peptides, was studied in a phase I trial of 22 patients with breast and ovarian cancer [23]. Toxicity was low, with one patient having a grade 3 injection site reaction and all other toxicities < grade 2. Twenty of the 21 patients developed FBP-specific lymphocytes and 16 had a sustained FBP-specific T-cell response 3 months after the vaccination series [23]. The second multi-peptide vaccine utilized five different HLA-A1/A2/A3 restricted peptides, including E39, derived from various antigens expressed on ovarian cancer cells. This vaccine was administered in a phase I trial to a total of 9 patients with Stage III/IV ovarian cancer [23]. Again, toxicity from this vaccine was very low with only < grade 2 toxicities. This vaccine was noted to be immunogenic, with production of peptide specific lymphocytes for each vaccine component and 8 of 9 patients developing peripheral blood lymphocytes specific for at least one component [24]. With regard to E39 specifically, this trial showed the E39 peptide to be safe, and confirmed its immunogenicity [24]. Both of these vaccines demonstrate the promise of a FBP-directed peptide vaccine by illustrating the excellent toxicity profile and the immunogenicity of the FBP in phenotypic and functional assays. The clinical efficacy of these vaccines has not been shown at this early stage of development, but evidence suggests they may have a role in treatment of these diseases at some point.

One major benefit of cancer vaccines, whether discussing the above vaccines, NeuVax, or E39, is how well they are tolerated. In our phase I/IIa trial of E39, we observed no grade 4 or 5 toxicities. The only grade 3 response was observed in the < 1000mcg cohort, indicating the increase in peptide dose did not cause any additional toxicity. In fact, we have previously shown through the use of GM-CSF as a control that the minimal toxicity associated with our vaccines is likely related to GM-CSF, not the peptide [24]. This may explain why our patients in the 1000mcg cohort experienced less toxicity than those in the < 1000mcg cohort, as the larger peptide dose may have resulted in increased consumption of GM-CSF at the site of injection, leaving less free GM-CSF to cause local or systemic toxicity. Given the severity of side effects associated with standard of care chemotherapy for endometrial and ovarian cancer patients, a low toxicity profile is key for any additional therapy given in the adjuvant setting. The E39 vaccine, even given at higher peptide dose, fulfills this requirement.

While a low toxicity profile is desirable in novel treatments, ultimately it is meaningless without signs of efficacy. Since the peptide vaccine strategy is based on stimulation of a robust T cell response that leads to cancer cell lysis, immune response is used as a marker of clinical response and has been positively correlated with clinical benefit by multiple investigators, particularly in the adjuvant setting [17, 26, 27]. Our data indicate that the E39 vaccine was able to effectively induce a robust immune response. Some patients did have a limited DTH response to E39 prior to vaccination, indicating previous exposure to FBP on the surface of their tumors, but vaccination increased this DTH response in all VG patients and increased the response to an even greater extent in patients receiving the 1000mcg dose, reaching statistical significance. Likely secondary to the limited number of patients in this trial, no definitive trend was appreciated that could relate increased DTH to decreased recurrence. The 1000mcg group, however, did have a greater increase in DTH from pre- to post-vaccination and the best clinical outcomes. Future trials will continue to use DTH as a surrogate for response and will seek to correlate DTH response to decreased recurrence of disease [17, 26, 27].

Although this trial was not powered to show a significant difference in overall recurrence rate, the vaccinated patients did see a non-statistically significant benefit. Moreover, in the planned subgroup analysis, patients receiving the 1000mcg dose of the vaccine had a pronounced benefit, reaching statistical significance when compared to both the CG and < 1000mcg group. The 1000mcg group was approximately five times less likely to experience a recurrence when compared to the CG. Given the high expression of FBP in endometrial and ovarian cancer, these patients were likely faced with significant previous exposure to the antigen, potentially leading to development of immunologic tolerance. In addition, they had undergone strong cytotoxic chemotherapeutic regimens prior to enrollment, potentially impairing the immune response to vaccination. As a result, this specific population may have required high doses of the peptide to mount a robust immune response and experience a clinical benefit.

Additional subset analysis assessing ovarian and endometrial cancer separately found that the 1000mcg group showed a statistically significant decrease in recurrence rate (compared to both the CG and < 1000mcg group) in the ovarian cancer patients, while the limited number of endometrial cancer patients receiving the 1000mcg prevented a meaningful statistical assessment. Finally, patients who presented with primary disease had much better treatment effect from the vaccine than those with recurrent disease. Limiting analysis to patients with primary disease, the 1000mcg group had a much lower risk of recurrence than the CG and < 1000mcg group. In recurrent patients, on the other hand, the 1000mcg group did not differ significantly from CG or < 1000mcg patients. These lessons will be incorporated into continued trials with E39, which will focus on patients with primary disease presentation, and will use the 1000mcg dose.

Given the promising results in this early trial, we are planning continued trials to examine the efficacy of E39. Our previous work with NeuVax has shown a sustained clinical benefit in patients receiving booster inoculations [19]. Based on these findings, this phase I/IIa trial of the E39 vaccine will be continued with a planned booster series involving the administration of two booster vaccinations to each disease free patient at 6 and 12 months after the PVS. Our primary analysis, to be conducted 24 months after completion of enrollment, will soon follow and further discuss our primary endpoint of 2-year DFS. Based on data from our primary analysis, a larger future phase IIb trial will be designed, utilizing the 1000mcg dose of E39+GM-CSF, including boosters, and enrolling disease free patients treated for primary ovarian and serous endometrial cancer.

## MATERIALS AND METHODS

### Patient characteristics and clinical protocols

This study is a prospective phase I/IIa dual-center trial being conducted under BB-IND #12391. Eligible patients were identified with primary or recurrent endometrial, ovarian, fallopian tube or peritoneal cancer and were disease-free after standard of care therapies. Patients were surgically or naturally post-menopausal. Exclusion criteria included patients currently receiving immunosuppressive therapy to include chemotherapy, steroids or methotrexate, poor health (ECOG > 2), evidence of end-organ dysfunction, pregnancy, breast feeding, history of autoimmune disease, and involvement in other experimental protocols (except with permission of the principal investigator of the other study).

Once enrolled, HLA status was evaluated to permit group assignment. E39 is an HLA-A2-restricted peptide (HLA-A2 is present in approximately 40-50% of the general population). HLA-A2 positive patients were thus inoculated with E39+GM-CSF, while HLA-A2 negative patients and those HLA-A2 positive individuals who declined vaccination were followed prospectively as matched controls for disease recurrence and progression. The clinical endpoints were long-term FBP immunity, recurrence rate, and 2-year DFS. This planned interim analysis was performed 12 months after completion of trial enrollment.

### Vaccine and vaccination series

The E39 peptide (GALE 301, FBP 191-199, EIWTHSYKV) was produced commercially by an FDA-compliant production facility for patient use. The peptide was purified to > 95% before use. Sterility, endotoxin (limulus amebocyte lysate test), and general safety testing was performed. In addition, the manufacturer performed purity/stability testing periodically. Single dose vials were tested for bacterial and fungal contaminants prior to use. The single dose vials were stored in the pharmacy at each institution. The bulk peptide was reconstituted to the following preparations: 100mcg/0.5mL, 500mcg/0.5mL, and 1000mcg/0.5mL. Each of these was mixed with 250mcg/1.0mL GM-CSF. This dose (250mcg) of GM-CSF has been previously determined to be a safe and effective dose, based on our prior work with NeuVax [7].

The combination of peptide and immunoadjuvant had a volume of 1.5mL, which was administered intradermally in 0.75mL inoculums at two different sites within 5cm of each other, ensuring these sites drained to the same nodal basin. The primary vaccination series (PVS) consisted of six total vaccinations, one given every 21-28 days, administered in the same lymph node draining area.

### Dosing

The phase I portion of this trial consisted of dose escalation to determine a safe and effective dose of the E39 peptide. The dose escalation scheme consisted of dosing cohorts of three patients receiving one the following doses: 100, 500, and 1,000 mcg of peptide, in addition to 250 mcg of GM-CSF. Prior to the fourth inoculation, each patient was assessed for liver, renal, and hematopoietic dysfunction. If organ function was stable and no dose limiting toxicity was observed, then the patient continued the series. After the third patient in a given dose group completed the third inoculation and organ function remained stable, the next dose group was initiated.

After completing the initial safety component of the trial, both 500 and 1000mcg dose cohorts were expanded. Patients receiving less than 1000mcg and those receiving 1000mcg were then compared for efficacy in the phase IIa component of the trial.

### Toxicity

Patients were monitored closely post-inoculation for an hour and re-examined 48-72 hours after each inoculation for local and systemic toxicity. The graded toxicity scale (NCI Common Terminology Criteria for Adverse Events, v4.03) was utilized to assess and grade local and systemic toxicities.

### *In vivo* immune monitoring

Patients were assessed for evidence of *in vivo* immunologic response by evaluation of delayed-type hypersensitivity (DTH) reaction, which was measured pre-vaccination and again post-PVS. A DTH response was assessed with 100 mcg of E39 (without GM-CSF) as well as a parallel control (equivalent volume of sterile saline) injected intradermally at a site on the posterior or anterior thigh on the opposite side from the vaccination site. The response was measured using the sensitive ballpoint-pen method in 2 dimensions at 48-72 hours post injection [16]. The orthogonal mean was determined for each DTH; its correlation to immunologic response has been validated and used in our previous work [17]. These values were compared between pre-PVS and post-PVS.

### Clinical recurrences of disease

Both the vaccinated patients and the observational control patients were monitored for evidence of clinical recurrence through the standard of care follow-up with their primary medical and/or surgical oncologist. This consisted of evaluations every three-months with clinical exams, laboratory tests and radiographic surveillance as indicated. Patients’ clinical records were assessed for evidence of clinical recurrence. DFS was measured from the date of enrollment. All patients were followed for clinical recurrence for up to two years at standard of care visits.

### Statistical analysis

A pre-specified, intention-to-treat analysis was performed at 12 months after the last patient was enrolled. Clinicopathologic data were compared between groups. Median and range were used to summarize continuous data and the groups were compared using a Mann-Whitney U test. Chi squared or Fischer exact test were used to compare categorical variables between groups. DTH data was presented as orthogonal means ± standard errors and compared using a Student's t test. Time to recurrence was measured from the date of enrollment. DFS was analyzed using the Kaplan-Meier method, and groups were compared using a simple log-rank test. Statistical analyses were performed using SPSS version 22 (IBM Corp. Released 2013. IBM SPSS Statistics for Windows, Version 22.0. Armonk, NY: IBM Corp.). Statistical significance was considered achieved if p < 0.05. Pre-specified subset analyses were also performed by dose cohort.

## CONCLUSIONs

In conclusion, this phase I/IIa trial in disease-free endometrial and ovarian cancer patients reveals that E39 + GM-CSF is a well-tolerated peptide vaccine, and elicits a strong, dose-dependent *in vivo* immune response with promising early efficacy in the 1000mcg dose cohort. This trial has established the peptide dose for a larger scale, prospective, randomized, GM-CSF controlled trial using the E39 peptide vaccine to prevent recurrence after treatment for primary disease in HLA-A2 positive endometrial and ovarian cancer patients.

This work was also partially funded by Galena Biopharma.

Funding sources were not involved with the study design; in the collection, analysis, or interpretation of data; in the writing of the report; or in the decision to submit the paper for publication.

## SUPPLEMENTARY MATERIALS TABLE


