# Supplementary material for: Interim analysis of a phase I/IIa trial assessing E39+GM-CSF, a folate binding protein vaccine, to prevent recurrence in ovarian and endometrial cancer patients
Source: Oncotarget. 2016 Nov 11;8(9):15912–23. doi: 10.18632/oncotarget.13305 (PMC5362533; doi:10.18632/oncotarget.13305)
Supplement: Supplementary file 1 [file oncotarget-08-15912-s001.pdf]

Interim analysis of a phase I/IIa trial assessing E39+GM-CSF, a folate binding protein vaccine, to prevent recurrence in ovarian and endometrial cancer patients

Supplementary Material

Supplemental Table 1. Tumor Characteristics

Histology	Subtype	Vaccinated (n=29)	Controls (n=22)
Endometrial Cancer - n(%)			
	Endometriod Adenocarcinoma	2 (6.9)	3 (13.6)
	Carcinosarcoma	1 (3.4)	0 (0.0)
	Papillary Serous Carcinoma	1 (3.4)	0 (0.0)
	Mixed Epithelial	1 (3.4)	0 (0.0)
Ovarian Cancer - n(%)			
	Serous Cystoadenocarcinoma	15 (51.7)	12 (54.5)
	Endometrioid Cystoadenocarcinoma	3 (10.3)	0 (0.0)
	Clear Cell Cystoadenocarcinoma	1 (3.4)	2 (9.1)
	Mixed Epithelial	1 (3.4)	0 (0.0)
	Papillary Serous Carcinoma	1 (3.4)	0 (0.0)
	Carcinosarcoma	0 (0.0)	2 (9.1)
	Mucinous Cystoadenocarcinoma	0 (0.0)	1 (4.5)
	Sex Cord Stromal	0 (0.0)	2 (9.1)
Primary Peritoneal - n(%)			
	Serous Cystoadenocarcinoma	2 (6.9)	0 (0.0)
Fallopian Tube - n(%)			

	Serous Cystoadenocarcinoma	1 (3.4)	0 (0.0)
